# Roles of necroptosis in alcoholic liver disease and hepatic pathogenesis

**DOI:** 10.1111/cpr.13193

**Published:** 2022-01-26

**Authors:** Ying Zhou, Ruoman Wu, Xinqi Wang, Xiaofeng Bao, Chunfeng Lu

**Affiliations:** ^1^ School of Pharmacy Nantong University Nantong Jiangsu China

## Abstract

Chronic alcohol consumption can cause alcoholic liver disease (ALD), leading to morbidity and mortality worldwide. Complex disease progression of ALD varies from alcoholic fatty liver to alcoholic steatohepatitis, eventually contributing to fibrosis and cirrhosis. Accumulating evidence revealed that necroptosis, a way of programmed cell death different from apoptosis and traditional necrosis, is involved in the underlying pathogenic molecular mechanism of ALD. Receptor‐interacting protein kinase 1 (RIPK1), RIPK3 and mixed‐lineage kinase domain‐like pseudokinase have been implicated as key mediators to execute necroptosis. Also, necroptosis has gained increasing attention due to its potential association with primary pathological hallmarks of ALD, including oxidative stress, hepatic steatosis and inflammation. This review summarizes the recent progress on the roles and mechanisms of necroptosis and focuses on the crosstalk between necroptosis and the other pathogenesis of ALD, providing a theoretical basis for targeting necroptosis as a novel treatment for ALD.

## Graphical abstract

Necroptosis has been implicated as a novel type of programmed cell death, which is mediated by RIPK1, RIPK3 and MLKL. When caspases are inhibited, RIPK1 interacts with RIPK3 to form a complex called ‘necrosome’ and initiates necroptosis. The activation of RIPK1 and RIPK3 is modulated by post‐translational modifications, including ubiquitination and phosphorylation, which is essential to control necroptosis

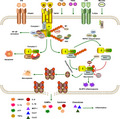

## 1 INTRODUCTION

Alcoholic liver disease (ALD) is a prevalent healthcare problem that leads to serious liver damage.^1^, ^2^ Steatosis is the earliest response to heavy drinking, featured by lipid accumulation in hepatocytes. Approximately 8%–20% of chronic heavy drinkers with steatosis will further progress to alcoholic steatohepatitis in the mediate stage, a more severe, inflammatory type of liver injury, which in turn contributes to liver fibrosis and liver cirrhosis in the advanced stage. Fibrosis, a wound healing response resulting from liver injury, is characterized by activation of hepatic stellate cells (HSCs) and excessive deposition of extracellular matrix proteins. The continuous wound healing response results in liver cirrhosis, 3%–10% of which will eventually develop into hepatocellular carcinoma.^2^

Necroptosis has been shown as a novel type of programmed necrotic cell death, which is mediated by receptor‐interacting protein kinase 1 (RIPK1), RIPK3 and mixed‐lineage kinase domain‐like pseudokinase (MLKL). When caspases are inhibited, RIPK1 interacts with RIPK3 to form a complex called ‘necrosome’ and initiates necroptosis. Necrotic cells release large amounts of cellular contents named damage‐associated molecular patterns (DAMPs), which trigger and amplify the inflammatory response. Previous studies have shown that lacking RIPK3 can effectively prevent alcohol‐induced liver damage, implying the relationship between necroptosis and ALD.^3^ Oxidative stress, lipid metabolism disorders, liver inflammation and cell death are the key drivers of alcohol‐induced liver injury.^4^ Mouse model studies have illustrated that necroptosis plays a crucial role in oxidative stress, steatosis and inflammation.^5^, ^6^, ^7^ This review discusses the mechanisms controlling necroptosis and the potential role of necroptosis in ALD, focusing particularly on its relevance to ALD‐associated pathological conditions.

## 2 MOLECULAR MECHANISMS UNDERLYING NECROPTOSIS

Cell death can be loosely categorized as apoptosis and necrosis. Apoptosis, a type of cell death dependent on the caspases, is characterized by nuclear lysis, chromatin condensation and cell shrink. Apoptotic cells can be dismantled into membrane‐wrapped fragments called apoptotic bodies that are immediately engulfed by surrounding macrophages, which typically are not perceived as a threat by the immune system.^8^ By contrast, necrosis is believed to be an ‘accidental’ type of cell death, featured by cell swelling, membrane burst and the release of intracellular components, including a class of molecules called DAMPs.^9^ DAMPs are defined as hallmarks of damage to trigger inflammatory responses.^10^

**FIGURE 1:**
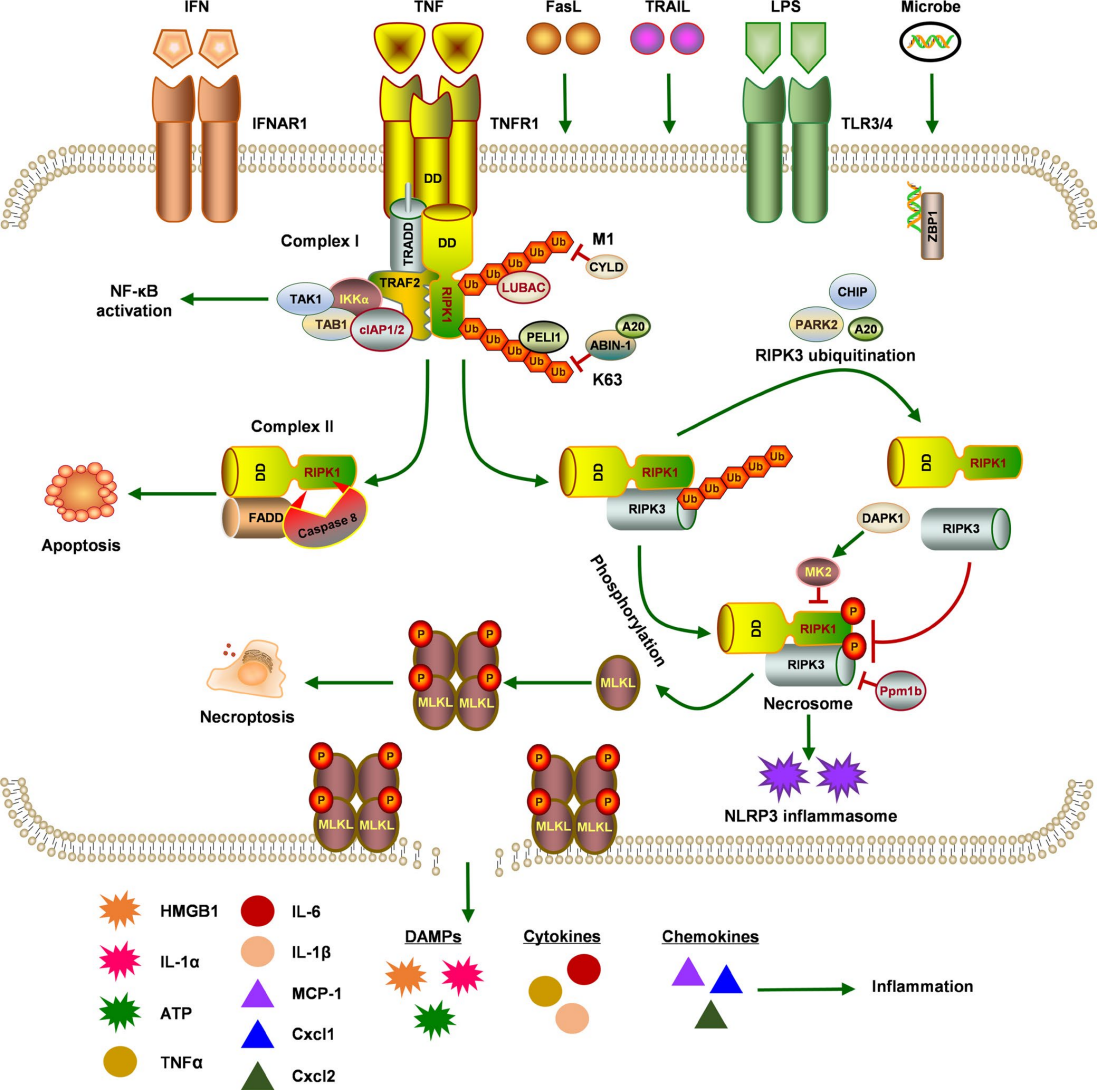
Molecular mechanisms of TNF‐induced necroptosis. A variety of signals may initiate necroptosis, and tumour necrosis factor (TNF) remains the most principal trigger of necroptosis. Upon stimulation of TNF, TNFR1 recruits TRADD and binds with RIPK1 to form complex I, including TRAF2, cIAP1/2, IKK and TAK1 complexes. In complex I, the M1‐Ubi of RIPK1 activates NF‐κB signalling pathway, which is regulated by M1‐Ubi enzyme complex LUBAC and the deubiquitinating complex CYLD. On the contrary, K63 ubiquitination mediates RIPK1 activation and subsequent cell death signalling, which is inhibited by ABIN‐1 and A20 or facilitated by PELI1. Activated RIPK1 connects with FADD and caspase‐8 to assemble into complex II, where caspase‐8 cleaves RIPK1 and triggers apoptosis. When caspase‐8 is inhibited, RIPK1 interacts with RIPK3 to form the RIPK1‐RIPK3 complex. Multiple ubiquitination of RIPK3 modulated by A20, CHIP and PARK2 can suppress RIPK1‐RIPK3 complex formation. Furthermore, RIPK1 and RIPK3 phosphorylate each other and create complex called ‘necrosome’. In necrosome, MK2 and DAPK1 act as negative regulators of RIPK1 to dampen necroptosis, and Ppm1b is identified as a RIPK3 phosphatase that prevents necroptosis through dephosphorylating RIPK3. During necroptosis, RIPK3 phosphorylates MLKL, which triggers its oligomerization and conveys to plasma membrane to form defined pores that induce plasma membrane permeabilization, resulting in the release of cellular contents including damage‐associated molecular patterns (DAMPs), cytokines and chemokines to execute inflammation

Necroptosis, a regulated cell death mediated by RIPK1 and RIPK3, shares a mechanistic similarity to apoptosis and bears a morphological resemblance to necrosis. RIPK1 and RIPK3 have a RIP homotypic interaction motif (RHIM), through which they interact with other RHIM domain‐containing proteins [RIPK1, RIPK3, Toll/IL‐1 receptor (TIR) domain‐containing adaptor‐inducing interferon‐β (TRIF) and Z‐DNA binding protein 1 (ZBP1)].^11^, ^12^ Death ligands [tumour necrosis factor (TNF), TNF‐related apoptosis‐inducing ligand (TRAIL) and Fas ligand (FasL)], interferons, Toll‐like receptor (TLR3/4) ligands, ZBP1,^13^, ^14^ Dectin‐1,^15^ inflammasome complex^16^ and microbial infection^17^, ^18^ can activate RIPK1 and RIPK3. Although a variety of signals may initiate necroptosis, tumour necrosis factor‐α (TNFα)‐induced necroptosis has been well illustrated. Therefore, take the TNF‐α signalling pathway as an example to introduce the mechanism of necroptosis (Figure 1).

Under the stimulation of TNF‐α, RIPK1 combines with TNFR‐associated death domain (TRADD) through its C‐terminal death domain (DD) to form complex I. This complex also includes cellular inhibitors of apoptosis protein 1 (cIAP1), cIAP2 and TNFR‐associated factor 2 (TRAF2).^19^, ^20^, ^21^ In complex I, IκB kinase (IKK) and TGF‐β‐activated kinase 1 (TAK1) complexes are recruited by ubiquitinated RIPK1, which in turn activates nuclear factor‐κB (NF‐κB) and mitogen‐activated protein kinase (MAPK)–mediated survival signalling pathways, inducing the expression of cytokines and chemokines.^22^ When RIPK1 is deubiquitinated, the permanent activation of NF‐κB signalling is limited and cells tend to the death pathway.^23^ Then, a cytosolic compound called complex II is formed, comprising kinase‐active RIPK1, caspase‐8 and FADD, which mediates apoptosis or interacts with RIPK3 to execute necroptosis.^24^, ^25^ In complex II, active caspase‐8 cleaves RIPK1 to be inactive, which initiates the pro‐apoptotic caspase activation cascade to enforce the apoptotic pathway.^26^ However, cell death shifts from apoptosis to necroptosis under low caspase‐8 activity conditions by drugs or genetic interventions. The interaction between RIPK1 and RIPK3 by virtue of their RHIM mediates the formation of a complex called ‘necrosome’, an oligomeric cytoplasmic complex, in which RIPK1 and RIPK3 phosphorylate each other and the latter recruits and phosphorylates MLKL.^19^, ^27^ Phosphorylated MLKL assembles into higher‐order oligomers and then translocates to the plasma membrane, eventually leading to membrane permeabilization, and cellular contents release by forming defined pores, resulting in the cell death.^28^, ^29^ A recent study proposed that MLKL and tight junction proteins are actively co‐transported from the necrosome to plasma membrane where they stably co‐accumulate as micron‐sized hot spots via Golgi‐, actin‐ and microtubule‐dependent mechanisms. In addition, phosphorylated MLKL accumulates at intercellular junctions, which aggravates necroptosis between neighbouring cells.^30^

## 3 POST‐TRANSLATIONAL MODIFICATIONS OF NECROPTOSIS

Accumulating evidence indicated that the activation of RIPK1 and RIPK3 is regulated by post‐translational modifications, consisting of ubiquitination and phosphorylation, which is essential to control necroptosis. Seven possible types of homotypic linkage (K6‐, K11‐, K27‐, K29‐, K33‐, K48‐ and K63‐linked ubiquitination) and linear ubiquitin linkage (also known as M1‐Ubi) have been confirmed. Ubiquitination mediates multiple cellular functions including protein degradation, receptor trafficking and protein–protein interactions.^31^ Similar to ubiquitination, phosphorylation has been established to be an important cellular event in numerous physiological processes. Most importantly, phosphorylation is indispensable for regulating protein stability, cellular localization and molecular interactions in protein complexes.^32^

### 3.1 The regulation of RIPK1 ubiquitination

TNF‐α stimulation induces TNFR1 trimerization to promote the formation of an intracellular complex named complex I, which recruits TRADD and binds with RIPK1 through its C‐terminal DD. RIPK1 is modified by different types of ubiquitination, comprising M1 and K63 ubiquitination, which are critical for regulating its activation.^33^, ^34^ Linear ubiquitin chain assembly complex (LUBAC) that contains haem‐oxidized IRP2 ubiquitin ligase 1L (HOIL‐1), HOIL‐1–interacting protein (HOIP) and Shank‐associated RH domain‐interacting protein (SHARPIN) mediates M1 ubiquitination of RIPK1 in complex I, where deubiquitinating enzyme cylindromatosis (CYLD) is recruited together.^35^, ^36^ Spermatogenesis‐associated 2 (SPATA2), an adaptor protein, bridges CYLD with HOIP to modulate the interaction between LUBAC and CYLD, which disassembles M1 ubiquitination.^37^, ^38^ The activation of RIPK1 is regulated by both the M1‐Ubi enzyme complex LUBAC and the deubiquitinating complex CYLD/SPATA2.^33^, ^34^ The M1‐Ubi of RIPK1 mediates the activation of NF‐κB signalling pathway and cell survival, while CYLD promotes RIPK1 activation by removing M1‐Ubi, leading to the initiation of TNF‐α–induced necroptosis.^33^

A20‐binding inhibitor of nuclear factor κB (ABIN‐1), a ubiquitin‐binding protein, is recruited rapidly into complex I in a manner that depends on M1‐ubiquitinating complex LUBAC to control the recruitment of A20, which is a deubiquitinating enzyme that modulates K63 ubiquitination of RIPK1.^39^ The phosphorylation of A20 has been demonstrated to enhance its deubiquitination activity.^40^ In TNF‐α–induced necroptosis, deficiency of ABIN‐1 advances K63 ubiquitination and activation of RIPK1 by impairing the recruitment of A20, resulting in cells succumbing to necroptosis, which emphasizes that the ABIN‐1/A20 axis limits the kinase activity of RIPK1 by regulating the K63 deubiquitination of RIPK1.^34^, ^41^ Opposed to ABIN‐1, Pellino 1 (PELI1) activates K63 ubiquitination of RIPK1 on K115, which facilitates the formation of necrosome and execution of necroptosis. Interestingly, PELI1 is required for promoting the combination of activated RIPK1 with its downstream molecular RIPK3 and the subsequent activation of RIPK3 and MLKL, while PELI1 deficiency does not affect RIPK1 activation.^42^ In summary, the activation of RIPK1 and the initiation of necroptosis depend on the K63 ubiquitination of RIPK1, which is regulated by multiple ubiquitinating and deubiquitinating enzymes.

### 3.2 The regulation of RIPK3 ubiquitination

Previous research highlights that K63 polyubiquitination of RIPK3 is indispensable for the formation of the RIPK1‐RIPK3 complex. A20 restricts the interaction between RIPK1 and RIPK3, which in turn inhibits RIPK3‐dependent necroptosis.^43^ Carboxyl terminus of Hsp70‐interacting protein (CHIP) performs the important function that mediates ubiquitylation of RIPK3 contributing to its lysosomal degradation, which blocks the development of TNF‐α–mediated necroptosis.^44^ Furthermore, RIPK1 expression is also negatively modulated by CHIP‐mediated ubiquitylation, implying that CHIP has emerged as a suppressor of the RIPK1‐RIPK3 complex formation. Parkin, also known as PARK2, prevents the assembly of the RIPK1‐RIPK3 complex by mediating the K33 polyubiquitination of RIPK3.^45^ AMP‐activated protein kinase (AMPK) phosphorylates and activates Parkin, which demonstrates that the AMPK/Parkin axis negatively regulates necroptosis via inhibiting the RIPK1‐RIPK3 complex formation. Taken together, these results indicate that diverse ubiquitination of RIPK3 modulates the formation of the RIPK1‐RIPK3 complex, restricting the process of necroptosis.

### 3.3 The regulation of RIPK1 phosphorylation

TNF‐α stimulation causes the activation of NF‐κB, p38 (MAPK) and its downstream kinase MAPK‐activated protein kinase 2 (MK2). Previous studies have shown that RIPK1 is the direct substrate of MK2 in TNF‐α signalling.^46^, ^47^ MK2 phosphorylates RIPK1 on S321 and S336, which dampens the cytoplasmic activation of RIPK1 and subsequent assembly of the RIPK1‐RIPK3 complex, also known as the necrosome, eventually suppressing RIPK1‐dependent necroptosis.^46^ Another research indicates that the phosphorylation of RIPK1 by MK2 at residue S321 diminishes its capability of binding FADD/caspase‐8, which in turn prevents TNF‐α–induced necroptosis.^47^ In addition, death‐associated protein kinase 1 (DAPK1) is identified as a negative regulator of necroptosis, deficiency of which enhances the formation of the RIPK1‐RIPK3‐MLKL complex, associated with reduced MK2‐mediated phosphorylation of RIPK1 on S321. DAPK1 binds with p38 (MAPK) and promotes its activation, which leads to elevated phosphorylation of MK2, resulting in the suppression of necroptosis.^48^ Together, the above researches illustrate that MK2 inhibits necroptosis through phosphorylating RIPK1 on S321 and S336, with consequently decreased activation of RIPK1 and reduced formation of necrosome.

### 3.4 The regulation of RIPK3 phosphorylation

The autophosphorylation of RIPK3 on Thr 231 and Ser 232 in the necrosome plays a significant role in triggering necroptosis. Recently, protein phosphatase 1B (Ppm1b) has been identified as a RIPK3 phosphatase that suppresses necroptosis through dephosphorylating RIPK3, which in turn blocks the recruitment of MLKL to the necrosome.^49^ Taken together, RIPK3 phosphorylation is essential for the interaction between RIPK3 and MLKL.

## 4 ROLES OF NECROPTOSIS IN ALCOHOLIC LIVER DISEASE

Alcoholic liver disease is featured by hepatic steatosis, inflammation and a large number of cell deaths, especially necroptosis. The relevance of necroptosis and ALD has been measured in different ALD models including chronic ethanol feeding, Gao‐binge ethanol feeding and ethanol gavage models. RIPK3, the key molecule of necroptosis signalling, was induced in murine models after ethanol exposure.^3^, ^50^, ^51^, ^52^ The biopsy samples from ALD and alcoholic liver cirrhosis patients showed increased RIPK3 and p‐MLKL expression, but not RIPK1, implying the important role of necroptosis in the occurrence and development of ALD.^3^, ^51^, ^53^, ^54^, ^55^

**FIGURE 2:**
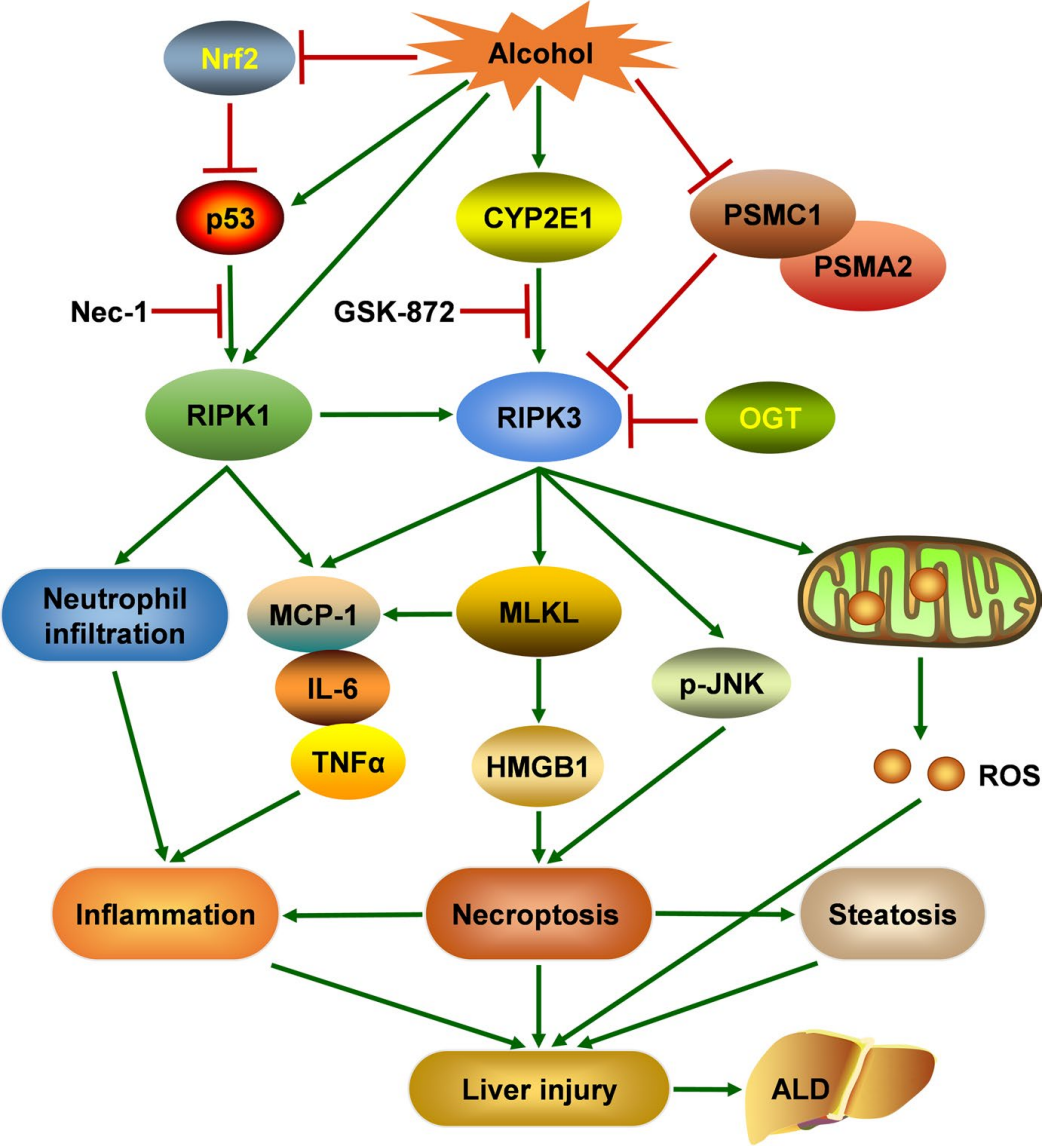
Relationship between necroptosis and alcoholic liver disease (ALD). Excessive alcohol consumption causes hepatic necroptosis, featured by increased expression of RIPK1 and RIPK3, which aggravates liver injury, steatosis and inflammation, eventually resulting in ALD. Elevated RIPK3 expression following alcohol exposure contributes to necroptosis, steatosis and mitochondrial ROS production, and RIPK1 activity is required for alcohol‐induced hepatic inflammation. Alcohol‐induced decreased expression of proteasome subunit α‐2 (PSMA2) and proteasome 26S subunit ATPase 1 (PSMC1) may lead to the hepatic accumulation of RIPK3. Furthermore, cytochrome P450 2E1 (CYP2E1) has been implicated as the upstream of RIPK3, while c‐jun N‐terminal kinase (JNK) acts as the downstream of RIPK3. O‐GlcNAc transferase (OGT) negatively regulates necroptosis via reducing the protein stability and expression of RIPK3. In addition, nuclear factor (erythroid‐derived 2)‐like 2 (Nrf2) pathway ameliorates alcohol‐induced liver injury by inhibiting necroptosis

Accumulating studies indicated that necroptosis makes a significant contribution to alcohol‐mediated liver injury, steatosis and inflammation (Figure 2). For instance, RIPK3 knockout (KO) had favourable effects on ethanol‐induced liver injury and steatosis, as measured by decreased aspartate aminotransferase (AST) and alanine aminotransferase (ALT) activity in plasma and reduced hepatic lipid accumulation following ethanol treatment compared with control mice.^3^ On the contrary, increased expression of RIPK1 in mouse liver was not detected in response to ethanol feeding, and the inhibition of RIPK1 by necrostatin‐1 (Nec‐1) did not reduce ethanol‐induced hepatocellular injury, indicating that ethanol‐induced liver damage is RIPK3‐dependent, rather than RIPK1‐dependent. The improvement in pro‐inflammatory mediators including TNF‐α, interleukin‐6 (IL‐6) and macrophage chemoattractant protein 1(MCP‐1) was observed in wild‐type (WT) mice during ethanol exposure, which was attenuated through RIPK3 deficiency. Another study used the chronic alcohol feeding plus acute binge model (Gao‐binge model) to investigate these findings. Consistent with the above studies, RIPK3 KO mice were more resistant to liver damage, steatosis and inflammation than WT mice during Gao‐binge alcohol treatment. However, the absence of RIPK3 failed to protect against hepatic neutrophil infiltration in response to Gao‐binge alcohol. Interestingly, inhibiting RIPK1 reduced alcohol‐induced liver steatosis, inflammatory gene expression and neutrophil recruitment.^51^ Therefore, we believe that RIPK3 accumulation following alcohol exposure contributes to cell death and steatosis, and RIPK1 activity is required for alcohol‐induced hepatic inflammation. Considering the role of RIPK1 and RIPK3 had been assessed, a recent study tested the hypothesis that MLKL, the downstream mediator of RIPK3, is implicated in ethanol‐induced liver injury. In contrast with the hypothesis, lack of MLKL did not protect mice from Gao‐binge ethanol or chronic ethanol–induced liver injury. Compared with WT mice, the expression of inflammatory cytokines after Gao‐binge ethanol feeding was decreased in MLKL^−/−^ mice, whereas the expression of chemokines was independent of MLKL genotype, as detected by chemokine (C‐X‐C motif) ligand 1 (Cxcl1) and Cxcl2 expression. Similarly, Gao‐binge ethanol–induced neutrophil accumulation was not ameliorated by MLKL knockout.^54^ In addition, Gao‐binge feeding–stimulated cytochrome P450 2E1 (CYP2E1) induction and endoplasmic reticulum (ER) stress were not affected by MLKL deletion, indicating that MLKL is not critical for ethanol‐induced liver injury.

Although RIPK3 KO seems to protect against ethanol‐induced liver damage, its underlying mechanism remains unclear. Previous research suggested that CYP2E1 made a significant contribution to RIPK3 expression, and phosphorylated c‐jun N‐terminal kinase (p‐JNK) was regulated by RIPK3 in mice during ethanol exposure.^3^ Deficiency of RIPK3 reduced ethanol‐induced p‐JNK expression, which evaluated that JNK acts as the downstream of RIPK3. The absence of CYP2E1 reversed ethanol‐mediated RIPK3 expression, whereas RIPK3 depletion did not alter the elevated expression of CYP2E1 induced by ethanol treatment, suggesting that CYP2E1 acts as the upstream contributor of RIPK3.^3^, ^51^ Wang et al. reported that RIPK3 expression is post‐translationally regulated in ethanol‐mediated liver injury, because the hepatic mRNA levels of RIPK3 were not altered by ethanol induction. Gao‐binge alcohol treatment diminished the protein levels of the proteasome subunit α‐2 (PSMA2) and the proteasome 26S subunit ATPase 1 (PSMC1) compared with pair‐fed mice, resulting in the decline of hepatic proteasome function. Meanwhile, liver‐specific PSMC1 KO mice had markedly increased RIPK3 expression. More importantly, the expression of PSMA2 and PSMC1 in the liver of ALD patients was decreased compared with the liver of healthy people.^51^ Therefore, we believed that alcohol‐induced impaired hepatic proteasome function might lead to the hepatic accumulation of RIPK3, contributing to necroptosis and steatosis. A recent study indicated that O‐linked β‐N‐acetylglucosamine (O‐GlcNAc) modification could moderate hepatic necroptosis and alcoholic liver fibrosis. Damaged O‐GlcNAc levels were observed in patients with alcoholic liver cirrhosis and in mice following alcohol exposure.^55^ Liver‐specific O‐GlcNAc transferase (OGT)‐KO mice (OGT‐LKO) emerged liver injury and liver fibrosis, and increased levels of pro‐inflammatory cytokines. OGT deficiency upregulated the expression of RIPK3 and MLKL and promoted the phosphorylation of RIPK3 and MLKL, whereas the protein levels of RIPK1, cleaved caspase‐3 and cleaved caspase‐8 were unchanged. Interestingly, Nec‐1 treatment significantly improved liver injury in OGT‐LKO mice. Consistent with experiments in mice, OGT‐deficient hepatocytes exhibited excessive necroptosis, which was blunted by a selective RIPK3 inhibitor GSK‐872. In addition, RIPK3 suppression with GSK‐872 alleviated mitochondrial ROS production induced by OGT deletion, which was associated with necroptosis downregulation. Eventually, Zhang et al. dissected the molecular mechanisms that OGT negatively regulates necroptosis via reducing the expression and protein stability of RIPK3, indicating OGT is a crucial inhibitor of necroptosis.

Furthermore, two studies reported the critical role of nuclear factor (erythroid‐derived 2)‐like 2 (Nrf2) signalling pathway in regulating necroptosis caused by ethanol.^52^, ^56^ In one research, curcumin inhibited necroptosis via the Nrf2/p35 pathway to ameliorate ethanol‐induced liver injury.^52^ Similarly, in the other research, ethanol exposure increased RIPK1 and RIPK3 expression and elevated DAMPs release, which were abrogated by gallic acid treatment through activating Nrf2.^56^

## 5 CROSSTALK BETWEEN NECROPTOSIS AND OTHER ALD‐ASSOCIATED PATHOGENESIS

It has been accepted that necroptosis has deleterious consequences in ALD. Apart from necroptosis, there are numerous pathological conditions contributing to ALD, including oxidative stress, hepatic steatosis and inflammation.^1^ Furthermore, emerging researches suggested the inseparable relationship between necroptosis and other pathological processes of ALD, providing new sights into targeting necroptosis for governing ALD‐associated pathogenesis, consequently as a novel approach to treating ALD.

### 5.1 Necroptosis and oxidative stress

Oxidative stress, which is induced by excessive reactive oxygen species (ROS) production, plays a vital role in liver damage. It is well known that accumulating ROS represents a crucial regulator of necroptosis, especially mitochondrial ROS produced by TNF‐α. Furthermore, necroptosis is indispensable to control the generation of ROS, which in turn exacerbates hepatocyte injury caused by oxidative stress (Table 1).

**TABLE 1:** Necroptosis and oxidative stress

Target gene	Animal model	Cell model	Drugs used	Overall outcome	Reference
RIPK1	APAP‐induced acute liver injury	APAP‐treated primary hepatocytes	Nec−1 (12.5 mg/kg)	RIPK1 inhibition reduced the production of APAP‐induced ROS and ameliorated hepatocyte damage caused by extracellular ROS	^57^
RIPK3	HFD model (16 weeks)	Palmitate‐treated AML−12 cells and primary hepatocytes	No data	RIPK3 knockdown alleviated HFD‐induced oxidative stress in vivo and palmitate‐induced oxidative stress in vitro by Nrf2/HO−1 signalling	^58^
RIPK1 and RIPK3	HFCD diet (6 and 18 weeks) MCD diet (2 and 8 weeks)	TNF‐α induced hepatocyte necroptosis	Nec−1 (100 μM)	The absence of RIPK1 or RIPK3 ameliorated TNF‐α–induced ROS production, and RIPK3 deficiency improved MCD diet–induced oxidative stress	^59^
RIPK1 and RIPK3	CCl_4_‐induced fibrosis	Primary HSCs and LX2 cells	Nec−1 (50 μM) Curcumol (15, 30 and 60 mg/kg in mice) Curcumol (20, 30 and 45 μM in LX2 cells)	Curcumol activated the JNK signalling pathway and increased the production of mitochondrial ROS in HSCs via RIPK1/RIPK3‐dependent necroptosis	^60^
RIPK1 and RIPK3	No data	Primary HSCs from rat livers cultured 6 passages for activation	Nec−1 (2 μg/ml) Gallic acid (25, 50 and 75 μM)	Gallic acid–induced elevated oxidative stress upregulated the expression of RIPK3 in HSCs through calcium signalling	^61^
RIPK1, RIPK3 and MLKL	No data	CPF‐triggered oxidative stress in L8824 cells	No data	CPF triggered oxidative stress by regulating the PTEN/PI3K/AKT axis, eventually inducing necroptosis of fish hepatocytes	^62^
RIPK1, RIPK3 and MLKL	Cd‐exposed liver injury (150 mg/kg)	No data	Selenium yeast (0.5 mg/kg)	Cd induced hepatic oxidative stress and activated the MAPK pathway, leading to significant increases in MLKL, RIPK1 and RIPK3 expression, which was reversed by selenium yeast	^63^
RIPK1 and RIPK3	Chronic liver injury model Acute liver injury model ACLF model	LPS and D‐Gal–treated LO2 cells	Nec−1 (50 μM) YQJPF (14.3 and 28.6 g/kg in rats) YQJPF (10, 20 and 40 μg/ml in LO2 cells) Atractylone (5 μM)	YQJPF had protective effects on hepatocyte necroptosis through suppressing ROS signalling	^64^

Abbreviations: ACLF, acute‐on‐chronic liver failure; APAP, acetaminophen; cadmium; CCl_4_, carbon tetrachloride; Cd; CPF, chlorpyrifos; D‐Gal, D‐galactosamine; HFCD, high‐fat choline‐deficient; HFD, high‐fat diet; HO‐1, haem oxygenase‐1; HSCs, hepatic stellate cells; LPS, lipopolysaccharide; MCD, methionine and choline‐deficient; MLKL, mixed linage kinase domain‐like; Nec‐1, necrostatin‐1; Nrf2, nuclear factor‐erythroid 2–related factor 2; RIPK1, receptor‐interacting protein kinase 1; RIPK3, receptor‐interacting protein kinase 3; ROS, reactive oxygen species; TNF‐α, tumour necrosis factor alpha; YQJPF, Yi‐Qi‐Jian‐Pi Formula.

Necroptosis is arising as the contributor and mediator of oxidative stress in a variety of liver diseases. Acetaminophen (APAP) induced hepatocyte ROS production and mitochondrial dysfunction, which was demonstrated to facilitate the development of hepatic injury, whereas RIPK1 inhibition by Nec‐1 protected hepatocytes from APAP‐damaged cellular and mitochondrial ROS induction. RIPK1 inhibition also enhanced the resistance of hepatocytes to oxidative stress and alleviated hepatocyte damage resulting from extracellular ROS.^57^ Oxidative stress was stimulated in response to high‐fat diet (HFD) in mice or palmitate in hepatocytes, as demonstrated by the enhanced ROS generation, malondialdehyde (MDA), hydrogen peroxide (H_2_O_2_) and O^2−^ levels, and by the damaged total antioxidant capacity (TAC) contents, superoxide dismutase (SOD), catalase (CAT) and glutathione (GSH) activities, which were significantly blocked by RIPK3 knockdown. In addition, RIPK3 knockdown alleviated HFD‐induced oxidative stress in vivo and palmitate‐induced oxidative stress in vitro through activating nuclear factor‐erythroid 2–related factor 2/haem oxygenase‐1 (Nrf2/HO‐1).^58^ Another study confirmed the association between necroptosis and oxidative stress. The suppression of RIPK1 with Nec‐1 or RIPK3 with RIPK3 siRNA ameliorated ROS production in hepatocytes exposed to TNF‐α, and RIPK3 deficiency improved methionine and choline‐deficient (MCD) diet‐induced oxidative stress, which indicated that oxidative stress is a downstream event of necroptosis.^59^ Curcumol triggered the overproduction of ROS in hepatic stellate cells (HSCs), as evidenced by increased MDA levels, and decreased SOD and GSH contents in a dose‐dependent manner. Similarly, mitochondrial ROS generation and depolarization were strongly induced in curcumol‐treated HSCs, which was attenuated by RIPK3 depletion via activating the JNK signalling pathway.^60^ Furthermore, necroptosis was impaired by N‐acetyl‐L‐cysteine (NAC), a ROS scavenger, suggesting ROS production is essential for necroptosis in HSCs under curcumol treatment.

Oxidative stress has a significant impact on governing hepatic necroptosis. For instance, gallic acid–induced elevated oxidative stress upregulated the key necroptotic regulatory proteins TRADD and RIPK3 in HSCs through calcium signalling.^61^ A study has shown the effect of chlorpyrifos (CPF) on oxidative stress, including increased levels of ROS and MDA, decreased levels of TAC, and damaged capability of antioxidant enzymes through regulating the phosphatase and tensin homolog deleted on chromosome ten/phosphatidylinositol 3‐kinase/threonine kinase (PTEN/PI3K/AKT) axis, eventually inducing necroptosis of fish hepatocytes.^62^ Another study revealed that the treatment of chickens with cadmium (Cd) induced hepatic oxidative stress and activated the MAPK pathway, leading to significant improvement in MLKL, RIPK1 and RIPK3 expression. In addition, selenium yeast (SEY) protected against the Cd‐induced necroptosis via inhibition of oxidative stress and MAPK pathway in chicken liver.^63^ The latest research indicated that Yi‐Qi‐Jian‐Pi Formula (YQJPF) decreased hepatocyte necroptosis in the acute‐on‐chronic liver failure (ACLF) model, consisting of inhibiting the expression of RIPK1 and RIPK3 and blocking the formation of necrosome.^64^ Meanwhile, YQJPF reduced the level of ROS in hepatocytes, which had the same effect as selective RIPK1 inhibitor Nec‐1, suggesting that YQJPF inhibited necroptosis by reducing ROS signalling.

Oxidative stress and necroptosis can form a positive feedback loop. ROS production facilitates the activation of RIPK1, leading to the recruitment of RIPK3 and necroptosis.^65^ RIPK1 and RIPK3 can induce ROS generation. Furthermore, RIPK1‐ and RIPK3‐mediated ROS production reactivates necroptosis, forming a positive feedback loop.^66^

### 5.2 Necroptosis and steatosis

Accumulating evidence illustrated the relevance of key necroptosis signalling molecules including RIPK3 and MLKL, and hepatic steatosis. RIPK3 has been indicated to be a lipid metabolism regulator, differentially controlling liver steatosis. Besides RIPK3, MLKL is arising as a critical player in lipid accumulation (Table 2).

**TABLE 2:** Necroptosis and steatosis

Target gene	Animal model	Cell model	Drugs used	Overall outcome	Reference
RIPK3	HFD model (6 and 12 weeks)	Palmitic acid–treated AML−12 cells	Nec−1 (30 µM)	RIPK3 knockout increased hepatic steatosis caused by HFD	^67^
RIPK3	HFD model (12 weeks)	OA‐treated HepG2 cells	GSK′843 (5 µM)	Lacking RIPK3 exacerbated hepatic lipid deposition induced by HFD	^68^
RIPK3	HFD model (16 weeks)	Palmitate‐treated AML−12 cells	No data	RIPK3 knockdown improved metabolic syndrome and hepatic steatosis in HFD‐fed mice via the TLR−4/NF‐κB and Nrf2/HO−1 signalling pathways	^58^
RIPK3	HFD model (20 weeks)	Palmitate‐treated NCTC1469 cells	GSK′872 (3 µM) Fisetin (20, 40 and 80 mg/kg in mice) Fisetin (10, 20 and 40 μM in NCTC1469 cells)	Fisetin suppressed HFD‐triggered liver lipid accumulation through downregulating RIPK3 activation	^69^
RIPK3	HFCD diet (6 and 18 weeks) MCD diet (2 and 8 weeks)	No data	Nec−1 (100 μM)	RIPK3 deficiency ameliorated MCD diet–induced steatosis	^59^
RIPK3	CDAA (32 and 66 weeks)	No data	No data	Deletion of RIPK3 altered hepatic lipidome upon long‐term CDAA feeding through upregulating PPARγ	^70^
MLKL	HFD model (12 weeks) MCD diet (12 weeks)	OA‐treated HepG2 cells	NSA (2.5 μM)	MLKL inhibition had protective effects on lipid metabolic disorders initiated by HFD	^71^
RIPK1 and MLKL	HFD model (16 weeks)	FFAs treated AML−12 cells	RIPA−56 (300 mg/kg) NSA (20 μM)	Inhibition of RIPK1 mitigated steatosis irritated by HFD via a MLKL‐dependent mechanism	^72^

Abbreviations: CDAA, choline‐deficient L‐amino acid‐defined; FFAs, free fatty acids; HFCD, high‐fat choline‐deficient; HFD, high‐fat diet; HO‐1, haem oxygenase‐1; JNK, c‐jun N‐terminal kinase; MCD, methionine and choline‐deficient; MLKL, mixed linage kinase domain‐like; Nec‐1, necrostatin‐1; NF‐κB, nuclear factor‐κB; Nrf2, nuclear factor‐erythroid 2–related factor 2; NSA, necrosulphonamide; OA, oleic acid; PPARγ, peroxisome proliferator–activated receptor γ; RIPK1, receptor‐interacting protein kinase 1; RIPK3, receptor‐interacting protein kinase 3; TLR‐4, Toll‐like receptor 4.

Lack of RIPK3 can exacerbate hepatic lipid deposition induced by a high‐fat diet (HFD). HFD feeding promoted MLKL phosphorylation in mice, associated with increased RIPK3 expression. Liver steatosis was stimulated after HFD in wild‐type mice, which was aggravated in RIPK3^−/−^ mice.^67^ RIPK3 deficiency exacerbated HFD‐induced apoptosis compared with WT mice, suggesting that inhibition of necroptosis switched cell death from necroptosis to apoptosis, resulting in steatosis and liver injury. However, RIPK1 suppression with Nec‐1 or RIPK3 knockdown with siRNA protected cells from palmitic acid–induced cytotoxicity in AML‐12 cells, which indicated that this shift requires additional signals, such as increased oxidative stress or inflammation. Another study displayed the same results. RIP3KO mice showed increased hepatic lipid deposition in response to HFD compared with WT mice. During HFD feeding, the very‐low‐density lipoprotein (VLDL) secretion markers, comprising microsomal triglyceride transfer protein (MTTP), protein disulphide isomerase (PDI) and apolipoprotein B (ApoB) were further reduced in RIPK3 KO mice.^68^ Primary hepatocyte–deleted RIPK3 had more lipid droplets than primary hepatocytes with RIPK3 after oleic acid (OA) treatment, whereas hepatic lipid accumulation was abrogated by RIPK3 overexpression. Whether using GSK’843 to inhibit RIPK3 will produce similar results in HepG2 cells? However, increased lipid deposition and decreased expression of MTTP, PDI and ApoB were not discovered in HepG2 cells treated with GSK'843.

Although the above studies exhibited that RIPK3 deletion aggravates HFD‐induced liver steatosis, others demonstrated the opposite results. RIPK3 deficiency reversed HFD‐induced increase in body weight and the liver weight/body weight ratio. In addition, HFD‐induced lipid deposition in liver tissue was attenuated, and the high levels of TG and TC in serum and liver were significantly downregulated in RIPK3 knockdown (KD) mice. The absence of RIPK3 expression was also associated with a significant decrease in the expression of major fatty acid synthesis–related factors [acetyl‐CoA carboxylase‐α (ACCα), fatty acid synthase (FAS) and stearoyl‐CoA desaturase 1 (SCD‐1)] and fatty acid uptake molecules [cluster of differentiation 36 (CD36), fatty acid transport protein 1 (FATP1) and fatty acid binding protein 1 (FABP1)], and a significant increase in the expression of fatty acid β oxidation–related genes [carnitine palmitoyltransferase‐1 (CPT‐1α) and peroxisome proliferator‐activated receptor α (PPARα)].^58^ AML‐12 cells were used to verify in vitro, which was consistent with the experimental results in vivo. Furthermore, RIPK3‐mediated lipid deposition was regulated by TLR‐4/NF‐κB and nuclear factor‐erythroid 2–related factor 2/haem oxygenase‐1 (Nrf2/HO‐1) signalling pathways. Xu's study showed that fisetin (Fn) could ameliorate hepatic steatosis triggered by HFD. Fn effectively inhibited liver lipid accumulation by modulating lipid metabolism–related indicators, consisting of recovering fatty acid β oxidation and suppressing fatty acid synthesis. The potential advantages of Fn in the prevention of HFD‐stimulated lipid metabolism disturbance were controlled by TNF‐α/RIPK3 axis, and finally resumed steatohepatitis.^69^

Other animal models were also used to investigate the role of RIPK3 in regulating lipid accumulation. After 2 weeks of feeding with a methionine and choline‐deficient (MCD) diet, WT mice displayed accumulation of lipid droplets, and RIPK3 depletion did not affect it. However, in response to 8 weeks of feeding, the lipid droplets of RIPK3^−/−^ mice were small and dispersed, which proved that lack of RIPK3 attenuated steatosis caused by MCD diet in the later stage.^59^ Deletion of RIPK3 influenced hepatic lipidome induced by long‐term choline‐deficient L‐amino acid (CDAA) feeding through upregulating PPARγ.^70^

The correlation between RIPK3 and hepatic steatosis is well established, and RIPK1/MLKL axis appears to be a considerable pathway of hepatic lipid deposition. MLKL inhibition had protective effects on lipid metabolic disorder initiated by HFD. MLKL KO mice conferred resistance to steatohepatitis due to suppressing lipid de novo synthesis.^71^ As observed in MLKL KO mice, HepG2 cells treated with necrosulphonamide (NSA), an MLKL inhibitor, showed reduced lipid storage and decreased expression of sterol‐regulatory element binding protein 1c (SREBP1c) and SCD‐1 compared with control cells, which suggested that HFD‐stimulated lipid accumulation was abrogated by MLKL suppression. RIPK1 inhibition by RIPA‐56 reversed steatosis irritated by HFD, as evidenced by reduced hepatic triglyceride (TG) content. Primary human hepatocytes treated with free fatty acid were used to gain insight into the underlying mechanisms that the protective effect of RIPA‐56 was dependent on facilitating fatty acid β‐oxidation.^72^ Inhibiting MLKL, the downstream molecular of RIPK1, was sufficient to decrease triglyceride content in hepatocytes, suggesting that the RIPK1/MLKL axis controls hepatic lipid accumulation in response to HFD feeding.

### 5.3 Necroptosis and inflammation

There is increasing evidence that necroptosis is a major source of inflammation in various liver diseases. RIPK1 has been recognized as an active executor of necroptosis and inflammation, closely correlated with ALD. RIPK3 and MLKL promote the secretion of pro‐inflammatory mediators. Also, HMGB1 released by necroptosis exerts its role in modulating inflammatory signalling by activating immune cells such as macrophages (Table 3).

**TABLE 3:** Necroptosis and inflammation

Target gene	Animal model	Cell model	Drugs used	Overall outcome	Reference
RIPK1	HFD model (16 weeks)	No data	RIPA−56 (300 mg/kg)	RIPK1 inhibition by RIPA−56 reduced inflammation in HFD‐fed mice	^72^
RIPK1 and RIPK3	HFD model (14 weeks) followed by hepatic IR injury	Primary hepatocytes treated with PA, followed by H/R injury	Nec−1 (1.65 mg/kg) GSK′872 (1.9 mM/kg)	HFD mice had increased inflammatory response during IR injury, and necroptosis inhibitors could suppress inflammation signalling pathways	^73^
RIPK1 and MLKL	APAP‐induced acute liver injury (300 mg/kg)	No data	Nec−1 (7 mg/kg)	RIPK1 suppression by Nec−1 inhibited NLRP3 inflammation signalling and protected against APAP‐induced liver damage in mice	^74^
RIPK1	HFD model (24 weeks)	BMDMs or primary Kupffer cells treated with PA and LPS	No data	HFD feeding induced liver inflammation in WT mice, which was obviously alleviated in RIPK1 kinase‐dead (RIPK1^K45A/K45A^) mice	^75^
RIPK1	Sod1 KO model	No data	Nec−1s (10 mg/kg, i.p.) once followed by Nec−1 (2.5–5 mg/day) in drinking water for 25 days	Sod1 KO mice had increased inflammation compared with control mice, while inflammation was significantly reduced in response to Nec−1 treatment	^76^
RIPK1	LPS‐induced liver damage (20 mg/kg)	Mouse hepatocytes treated with 10% septic serum	miR−425‐5p agomiR (30 mg/kg) miR−425‐5p antagomiR (80 mg/kg)	miR−425‐5p negatively regulated RIPK1 expression to improve LPS‐induced liver inflammatory response	^77^
RIPK3	CDAA (32 and 66 weeks)	No data	No data	RIPK3 deficiency ameliorated hepatic inflammation induced by long‐term CDAA feeding	^70^
RIPK3	HFD model (16 weeks)	Palmitate‐treated AML−12 cells	No data	RIPK3 knockdown improved hepatic inflammation in HFD‐fed mice	^58^
MLKL	WD model (5, 8 and 12 weeks) followed by hepatic IR injury	No data	No data	MLKL knockout mice exhibited decreased hepatic neutrophil infiltration and inflammation and were protected from hepatic IR injury during HFD	^79^
HMGB1	Intestinal I/R model	No data	Nec−1 (1 mg/kg)	HMGB1 inhibition alleviated intestinal I/R‐associated hepatocyte necroptosis and tissue inflammation	^80^

Abbreviations: APAP, acetaminophen; CDAA, choline‐deficient L‐amino acid‐defined; HFD, high‐fat diet; HMGB1, high‐mobility group box‐1; H/R, hypoxia/ reperfusion; IR, ischaemia‐reperfusion; LPS, lipopolysaccharide; MLKL, mixed linage kinase domain‐like; Nec‐1, necrostatin‐1; NLRP3, pyrin domain‐containing protein 3; PA, palmitic acid; RIPK1, receptor‐interacting protein kinase 1; RIPK3, receptor‐interacting protein kinase 3; Sod1, Cu/Zn‐superoxide dismutase; TNF‐α, tumour necrosis factor alpha; WD, Western diet.

Receptor‐interacting protein kinase 1 suppression can attenuate the hepatic inflammatory response induced by a great deal of stimulation. For instance, the expression of TNF‐α and other inflammatory markers such as CC‐chemokine ligand 2 (CCL‐2), NOD‐like receptor (NLR) family, pyrin domain‐containing 3 (NLRP3), and interleukin‐1β (IL‐1β) was enhanced in the livers of HFD‐fed mice, which were abrogated after both prophylactic and curative treatment of RIPA‐56, a particular inhibitor of RIPK1. HFD‐aggravated mRNA levels of F4/80 and Mcp‐1, a potent macrophage chemoattractant, were decreased by downregulating RIPK1.^72^ Moreover, the inhibition of necroptosis by Nec‐1 and GSK’872 ameliorated the inflammatory signalling pathways activated during ischaemia‐reperfusion injury (IRI) in HFD mice, which was managed by transcription factors, including nuclear factor‐κB (NF‐κB), c‐jun N‐terminal kinase (JNK), extracellular‐regulated MAP kinase (ERK), mitogen‐activated protein kinase 14 (p38) and NF‐κB inhibitor alpha (IKBα).^73^ Recent research reported that the expression of critical molecules in NLRP3 inflammasome was significantly higher in APAP‐treated mice than in control mice, and this difference disappeared following inhibition of necroptosis with Nec‐1. In addition, further investigation indicated that the level of IL‐1β in mouse liver was closely consistent with the level of p‐MLKL in response to APAP exposure, as evidenced by co‐localization of p‐MLKL and IL‐1β.^74^ Tao's study demonstrated that RIPK1 activity was promoted in hepatic macrophages after HFD feeding, but hepatic inflammation was obviously alleviated in RIPK1 kinase‐dead (RIPK1^K45A/K45A^) mice compared with WT mice, implying that RIPK1 is required for controlling hepatic macrophage infiltration and activation.^75^ Also, the results obtained in both bone marrow–derived macrophages (BMDMs) and primary Kupffer cells (KCs) further confirmed the hypothesis that RIPK1 plays a crucial role in mediating inflammasome activity. Antioxidant enzyme Cu/Zn‐superoxide dismutase mice (Sod1^−/−^ mice) develop the non‐alcoholic fatty liver disease (NAFLD) with age. Expression of MLKL and RIPK3 and phosphorylation of MLKL and RIPK3 were extremely increased in the livers of Sod1KO mice compared with WT mice. Markers of pro‐inflammatory M1 macrophages, NF‐κB pathway, NLRP3 inflammasome and transcript levels of pro‐inflammatory cytokines and chemokines were perceptibly exacerbated. Treatment of Sod1KO mice with Nec‐1s reversed these conditions, suggesting that elevated necroptosis in the liver led to inflammation in Sod1KO mice.^76^ In addition, miR‑425‑5p could moderate inflammation in septic hepatocytes through targeting the 3’UTR of RIPK1 mRNA to dampen RIPK1‑mediated necroptosis.^77^

Consistent with RIPK1, RIPK3 has been regarded as an essential contributor to inflammation. For example, RIPK3 deficiency ameliorated CDAA‐induced liver inflammation.^70^ Elevated secretion of TNF‐α, IL‐6, IL‐1β, IL‐18 and CCL‐2 following HFD treatment was significantly attenuated by RIPK3 knockdown. Additionally, suppressing RIPK3 alleviated the expression of molecules implicated in NF‐κB and inflammasome pathways in HFD‐diet mice.^58^ O‐GlcNAc transferase (OGT) inhibited the innate immune activation and dampened septic inflammation via O‐GlcNAcylation of RIPK3 on threonine 467 (T467) to inactive RIPK3, resulting in preventing RIPK1‐RIPK3 interaction and avoiding downstream innate immunity and necroptosis signalling.^78^

Depletion of MLKL and HMGB1 has a protective effect against hepatic inflammation. MLKL deletion decreased hepatic neutrophil infiltration and inflammation caused by hepatic IR injury.^79^ Necroptosis was induced in liver after intestinal ischaemia/reperfusion in rats, and HMGB1 cytoplasm translocation was observed in hepatocytes after intestinal I/R challenge, which were markedly attenuated by Nec‐1 administration. HMGB1 inhibition with the neutralizing antibody and inhibitor alleviated I/R‐associated hepatocyte necroptosis and tissue inflammation, and HMGB1 suppression promoted circulating macrophages and hepatic KC M2 polarization, contributing to protecting against liver injury.^80^ Taken together, these findings demonstrated that necroptosis acts as an emerging regulator in inflammation, and inhibition markers of necroptosis can protect against hepatic damage caused by inflammatory stimulation.

## 6 CONCLUSIONS

The pathogenesis of alcoholic liver disease is very complex. A variety of pathological factors, including oxidative stress, hepatic steatosis and inflammation, are considered critical elements for liver damage in ALD. Recent advances reported that necroptosis is significantly implicated in the occurrence and development of ALD by activating RIPK1, RIPK3 and MLKL. In this review, we illustrated the underlying mechanisms of necroptosis and post‐translational modifications of necroptosis comprising ubiquitination and phosphorylation of RIPK1 and RIPK3. MLKL is the most downstream molecule of necroptosis. However, the mechanism that MLKL induces plasma membrane permeabilization still remains to be explicated. Besides their well‐established roles in necroptosis execution, the association between necroptosis regulators and the above pathological conditions in ALD has been confirmed. The absence of RIPK3 attenuated cell death, steatosis and mitochondrial ROS production, and RIPK1 deficiency dampened hepatic inflammation after alcohol exposure. Although necroptosis suppression seems to be beneficial, whether the results of mouse models are absolutely connected with human pathophysiology remains undefined. Therefore, further research is needed to explore their specific contribution to disease pathogenesis and examine inhibition of the interaction between necroptosis and pathological factors by pharmaceutical methods as potential therapeutic approaches to alleviating ALD.

## CONFLICT OF INTERESTS

The authors declare no conflicts of interest.

## AUTHOR CONTRIBUTIONS

C. Lu conceptualized the study. Y. Zhou, C. Lu, R. Wu, X. Wang and X. Bao contributed to literature retrieval and collection. Y. Zhou and C. Lu wrote and prepared the original draft. C. Lu wrote, reviewed and edited the manuscript. C. Lu. contributed to funding acquisition. All authors have read and agreed to the published version of the manuscript.

## Data Availability

Data sharing is not applicable to this article as no data sets were generated or analysed during the current study.
